# Predictors of renal infarction in patients presenting to the emergency department with flank pain: A retrospective observational study

**DOI:** 10.1371/journal.pone.0261054

**Published:** 2021-12-07

**Authors:** Sangun Nah, Sangsoo Han, Han Bit Kim, Sohyeon Chun, Sechan Kim, Seungho Woo, Ji Eun Moon, Young Soon Cho

**Affiliations:** 1 Department of Emergency Medicine, Soonchunhyang University Bucheon Hospital, Bucheon, Republic of Korea; 2 Department of Biostatistics, Clinical Trial Center, Soonchunhyang University Bucheon Hospital, Bucheon, Republic of Korea; Kaohsiung Medical University Hospital, TAIWAN

## Abstract

**Objectives:**

Flank pain is a common symptom in the emergency department and can be caused by a variety of diseases. Renal infarction (RI) is a very rare disease, and many RI patients complain of flank pain. However, there is no definitive predictor of RI when patients complain of flank pain. This study aimed to identify the clinical factors for predicting RI in patients with flank pain.

**Methods:**

This retrospective single-center study was conducted on patients complaining of flank pain from January 2016 to March 2020 at a South Korean tertiary care hospital. Exclusion criteria included patients who did not undergo contrast-enhanced computed tomography, age < 18 years, and trauma. Demographic and laboratory data were obtained from medical records. Logistic regression analysis was conducted to identify predictors of RI occurrence.

**Results:**

In all, 2,131 patients were enrolled, and 39 (1.8%) had RI. From a multivariable logistic regression analysis, an age ≥ 65 years (odds ratio [OR], 3.249; 95% confidence interval [CI], 1.366–7.725; *p* = 0.008), male sex (OR, 2.846; 95% CI, 1.190–6.808; *p* = 0.019), atrial fibrillation (OR, 10.386; 95% CI, 3.724–28.961; *p* < 0.001), current smoker (OR, 10.022; 95% CI, 4.565–22.001; *p* < 0.001), and no hematuria (OR, 0.267; 95% CI, 0.114–0.628; *p* = 0.002) were significantly associated with the occurrence of RI.

**Conclusions:**

Five clinical factors, i.e., age ≥ 65 years, male sex, atrial fibrillation, current smoker, and no hematuria, were significantly associated with the occurrence of RI in patients with flank pain.

## Introduction

Flank pain is a common symptom in the emergency department (ED) that can be caused by various urinary and extra-urinary diseases (e.g., urolithiasis, tumor, lower lobe pneumonia, urinary tract infection, and cholecystitis) [1]. Renal infarction (RI), a disease that can occur in patients complaining of flank pain, develops when the renal artery flow is abruptly blocked, and previous studies have reported that the incidence rate of RI is 0.004–0.007% in the ED and that 65–85% of RI patients complain of flank pain [2, 3]. RI occurs very rarely and is not easy to diagnose early because its symptoms are similar to those of other diseases mentioned above, such as urolithiasis [1, 4, 5]. According to previous studies, acute RI is often diagnosed accidentally when treating patients complaining of flank pain or with other diseases [1, 2, 4]. If the diagnosis of acute RI is missed initially or delayed, patients are at risk of renal failure, which can lead to death [1, 6–8]. Therefore, an early diagnosis of acute RI is essential for effective treatment and to preserve renal function without leaving any other sequelae [2].

Contrast-enhanced computed tomography (CT) is a non-invasive imaging diagnostic technique that helps to differentiate abscesses, biliary tract disease, tumors, cysts, gynecological diseases, and other abdominal diseases. In addition, according to previous studies, contrast-enhanced CT can be used to quickly diagnose acute RI even in ED patients complaining of non-specific symptoms [2, 9–11]. Therefore, contrast-enhanced CT has been suggested as the first choice for a radiological diagnosis in patients with suspected RI or patients with flank pain but an uncertain diagnosis [12].

The risk factors of acute RI have been suggested to include atrial fibrillation (AFib); a history of infarction, valvular, or ischemic heart disease; and a hypercoagulable state [2, 13]. RI can be suspected when hematuria or elevated lactate dehydrogenase (LDH) is observed [6]. However, due to the very low incidence of RI, all previous studies have been descriptive reports conducted only on RI patients, and the clinical characteristics of RI patients and non-RI patients were not compared or studied. Therefore, the purpose of this study was to confirm the predictors of RI in the ED by retrospectively analyzing the medical records of patients with flank pain and comparing the clinical features and diagnostic results of patients with and without RI.

## Materials and methods

### Study population

This retrospective observational study was conducted at a tertiary care hospital in Bucheon, South Korea. This study was approved by the Soonchunhyang University Bucheon Hospital institutional review board (IRB file no. 2020-11-003). And this study investigated only pre-existing data, so informed consent was not obtained from each patient. The study was conducted on patients who visited the Soonchunhyang University Bucheon Hospital ED from January 2016 to March 2020. Only patients who underwent contrast-enhanced CT among those who expressed “flank pain” as their chief complaint during the study period were included in the study. Those who did not undergo CT or who underwent non-contrast enhanced CT were excluded from the study. Also, patients < 18 years of age and patients with flank pain due to trauma were excluded.

### Data collection

Clinical, demographic, and laboratory data were obtained for each patient through retrospective chart review based on arrival at the ED. Contrast-enhanced CT was performed based on the judgment of the clinician regarding the patient’s condition. The estimated glomerular filtration rate (eGFR) was calculated using the Modification of Diet in Renal Diseases equation. Hematuria was defined as five or more erythrocytes per high power field (HPF), and pyuria was defined as 10 or more leukocytes per HPF, in accordance with previous studies [14, 15]. A current smoker was defined as a patient who had a current smoking habit or stopped smoking within 6 months before visiting the ED [16]. Fever was defined as a body temperature ≥ 37.8°C [17]. The RI diagnosis was confirmed by a radiologist’s official reading of the CT results. The CT findings of RI included the following: the presence of a wedge-shaped parenchymal perfusion defect with or without a cortical rim sign, without mass effect or major peri-renal stranding [2, 6].

### Data analysis

IBM SPSS statistics software, version 25 (IBM Corp., Armonk, NY, USA) and R ver. 3.5.3 software (The R Foundation for Statistical Computing, Vienna, Austria) were used for statistical analyses. Continuous variables were examined using the Shapiro-Wilk test and in a histogram and are expressed as the means ± standard deviations when the data had a normal distribution or as medians with interquartile ranges when the data did not have a normal distribution. Categorical variables are expressed as absolute numbers or percentages. We compared all variables by dividing the population into two groups according to the presence or absence of RI. Continuous variables were analyzed using Student’s *t*-test or the Mann-Whitney U test, and categorical variables were analyzed using Fisher’s exact test or the chi-square test. Variables with a *p*-value < 0.05 in the univariable analysis were included in as candidate variables for multivariable logistic regression analysis. Adjusted odds ratios (ORs) with 95% confidence intervals (CIs) were calculated using logistic regression analysis. The variability inflation factor was used to verify multi-collinearity. To investigate the effects of age, patients were divided into two groups: age < 65 years vs. age ≥ 65 years. Receiver operating characteristic curves were used for the multivariable logistic regression model.

## Results

**Fig 1:**
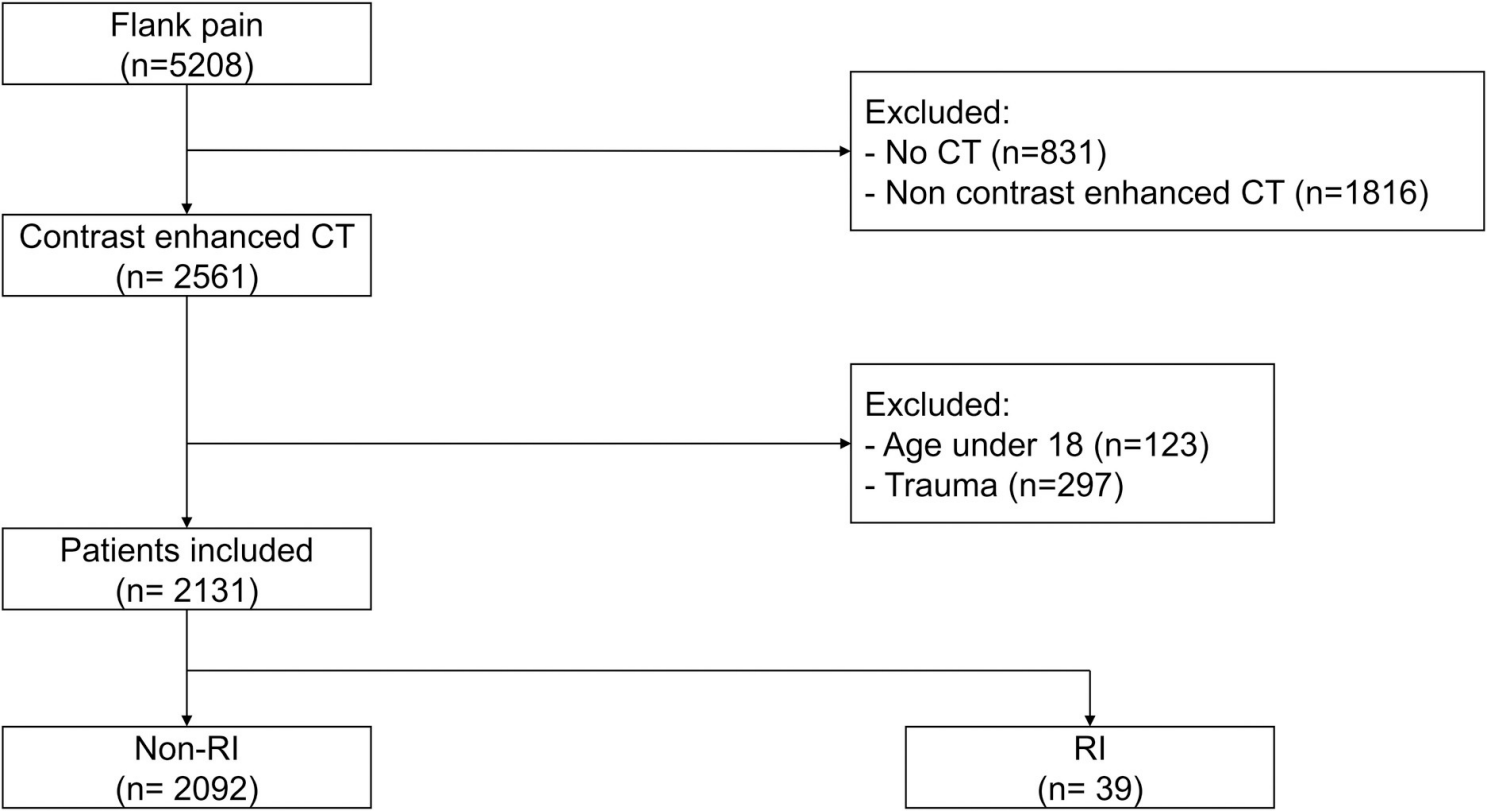
Flow chart of study subject enrollment. Abbreviations: CT, computed tomography; RI, renal infarction.

During the study period, 5,208 patients complaining of flank pain visited the ED. Of these, 2,131 patients were included in the final analysis after excluding 3,077 patients, and 39 (1.8%) were diagnosed with RI. The exclusion criteria were no CT data (n = 831), non-contrast enhanced CT (n = 1,816), age < 18 years (n = 123), and trauma (n = 297) (Fig 1).

Statistical comparisons of the non-RI and RI groups are presented in Table 1. The numbers of participants aged ≥ 65 years and males were 340 (16.3%) and 898 (42.9%) in the non-RI group and 20 (51.3%) and 29 (74.4%) in the RI group, respectively. The average systolic blood pressure and body temperature were, respectively, 133.9 mmHg and 36.9°C in the non-RI group, and 141.2 mmHg and 36.7°C in the RI group. The rate of comorbidities, such as hypertension, myocardial infarction, stroke, and AFib, was higher in the RI group than in the non-RI group. The current smoker percentage was 51.3% in the RI group and 6.7% in the non-RI group. The average eGFR value in the RI group was 58.9 mL/min/1.73 m^2^, which was lower than in the non-RI group (69.4 mL/min/1.73 m^2^). The percentage of patients with hematuria was 51.9% in the non-RI group and 23.1% in the RI group. The result of the baseline characteristics of patients according to sex is presented in S1 Table also.

**Table 1:** Comparison of the baseline characteristics between the two groups.

	Non-RI	RI	*p*-value
	(n = 2092)	(n = 39)	
Age, years	50.3 ± 15.5	58.97 ± 20.4	<0.01
Age≥65 (%)	340 (16.3)	20 (51.3)	<0.01*
Male, n (%)	898 (42.9)	29 (74.4)	<0.01**
BMI, kg/m^2^	24.2 ± 4.1	23.7 ± 4.8	0.56
Vital signs			
Systolic BP, mmHg	133.9 ± 20.5	141.2 ± 30.6	0.15
Diastolic BP, mmHg	82.3 ± 13.5	82.8 ± 17.1	0.78
Heart rate, beats/min	84.3 ± 13.5	86.6 ± 19.7	0.35
Respiratory rate, /min	19.5 ± 1.2	19.6 ± 1.7	0.37
Body temperature, °C	36.9 ± 0.8	36.7 ± 0.6	0.03
Comorbidities, n (%)			
HTN	561 (26.8)	21(53.8)	0.32*
DM	292 (14.0)	8 (20.5)	>0.99*
MI	46 (2.2)	6 (15.4)	<0.01**
Stroke	51 (2.4)	5 (12.8)	<0.01**
Cancer	166 (7.9)	6 (15.4)	0.13**
AFib	32 (1.5)	10 (25.6)	<0.01**
Current smoker, n (%)	141 (6.7)	20 (51.3)	<0.01*
Symptoms, n (%)			
Nausea	442 (21.1)	6 (15.4)	0.50*
Vomiting	191 (9.13)	3 (7.7)	>0.99**
Diarrhea	71 (3.4)	2 (5.1)	0.39**
Laboratory findings			
eGFR, mL/min/1.73m^2^	69.4 ± 16.5	58.9 ± 20.4	<0.01
White blood cells, ×10^3^/mm^3^	9.8 ± 4.6	11.1 ± 4.2	0.07
Platelet, ×10^3^/mm^3^	238.8 ± 87.0	229.1 ± 108.4	0.49
AST, U/L	21 [17–28]	43 [25.5–63.5]	0.17
ALT, U/L	20 [13–32]	29 [23–48.5]	0.40
CRP, mg/dL	0.2 [0.01–1.7]	0.5 [0.2–2.9]	0.84
Pyuria, n (%)	441 (21.1)	3 (7.7)	0.04*
Hematuria, n (%)	1085 (51.9)	9 (23.1)	<0.01*

Note: Values are presented as the means ± standard deviations, medians [interquartile ranges], or numbers (proportions). *Pearson’s χ^2^ test,

** Fisher’s exact test. Pyuria is defined as 10 or more white blood cells per high power field (HPF). Hematuria is defined as five or more erythrocytes per HPF.

Abbreviations: RI, renal infarction; BMI, body mass index; BP, blood pressure; HTN, hypertension; DM, diabetes mellitus; MI, myocardial infarction; AFib, atrial fibrillation; eGFR, estimated glomerular filtration rate; AST, aspartate transaminase; ALT, alanine transaminase; CRP, C-reactive protein.

**Fig 2:**
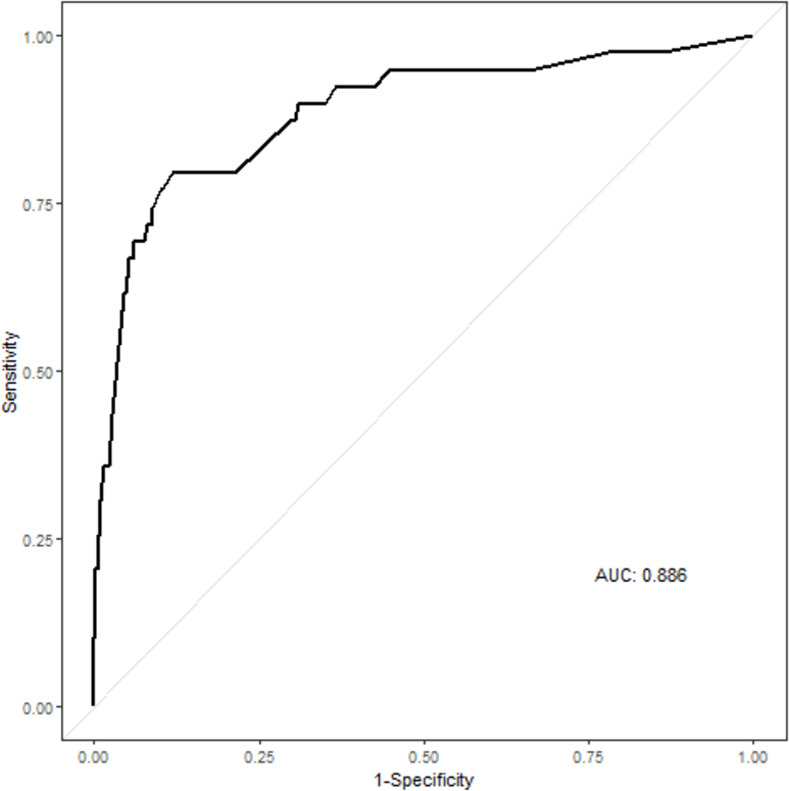
Receiver operating characteristic curves for the multiple logistic regression analysis model of risk factors for renal infarction. Abbreviations: AUC, area under curve.

The multivariable analysis was performed with significant (*p* < 0.05) variables from the univariable analysis (Table 2). The results of the multivariable analysis are presented in Table 3. The occurrence of RI was significantly associated with age ≥ 65 years (OR, 3.249; 95% CI, 1.366–7.725; *p* = 0.008), male sex (OR, 2.846; 95% CI, 1.190–6.808; *p* = 0.019), AFib (OR, 10.386; 95% CI, 3.724–28.961; *p* <0.001), current smoker (OR, 10.022; 95% CI, 4.565–22.001; *p* < 0.001), and no hematuria (OR, 0.267; 95% CI, 0.114–0.628; *p* = 0.002). The area under the curve for the multivariable logistic regression analysis was 0.886 (95% CI, 0.823–0.950) (Fig 2). Also, the result of the sex-stratified logistic regression analysis of predictors of RI is presented in S2 Table.

**Table 2:** Univariable logistic regression analyses of predictors of renal infarction.

	Univariable		
	OR	95% CI	*p*-value
Age (≥ 65 years)	5.421	2.863–10.266	<0.001
Male	3.853	1.868–7.946	<0.001
Body temperature (≥ 37.8°C)	0.409	0.098–1.708	0.220
Comorbidities			
HTN	3.182	1.683–6.016	<0.001
MI	8.083	3.229–20.235	<0.001
Stroke	5.882	2.210–15.658	<0.001
AFib	22.187	9.979–49.334	<0.001
Current smoker	14.558	7.594–27.907	<0.001
Laboratory findings			
eGFR (< 60 mL/min/1.73 m^2^)	3.001	1.590–5.665	0.001
Pyuria	0.312	0.096–1.017	0.053
Hematuria	0.278	0.131–0.589	0.001

Note: Pyuria is defined as 10 or more white blood cells per high power field (HPF). Hematuria is defined as five or more erythrocytes per HPF.

Abbreviations: OR, odds ratio; CI, confidence interval; HTN, hypertension; MI, myocardial infarction; AFib, atrial fibrillation; eGFR, estimated glomerular filtration rate.

**Table 3:** Multivariable logistic regression analysis of predictors of renal infarction.

	Multivariable			
	OR	95% CI	*p*-value	VIF
Age (≥ 65 years)	3.249	1.366–7.725	0.008	1.509
Male	2.846	1.190–6.808	0.019	1.278
Comorbidities				
HTN	1.112	0.486–2.542	0.801	1.390
MI	1.234	0.382–3.985	0.725	1.148
Stroke	1.265	0.368–4.351	0.709	1.194
AFib	10.386	3.724–28.961	<0.001	1.237
Current smoker	10.022	4.565–22.001	<0.001	1.241
Laboratory findings				
eGFR (< 60 mL/min/1.73 m^2^)	1.782	0.812–3.914	0.150	1.242
Hematuria	0.267	0.114–0.628	0.002	1.085

Note: Hematuria is defined as five or more erythrocytes per high power field.

Abbreviations: OR, odds ratio; CI, confidence interval; VIF, variance inflation factor; HTN, hypertension; MI, myocardial infarction; AFib, atrial fibrillation; eGFR, estimated glomerular filtration rate.

**Fig 3:**
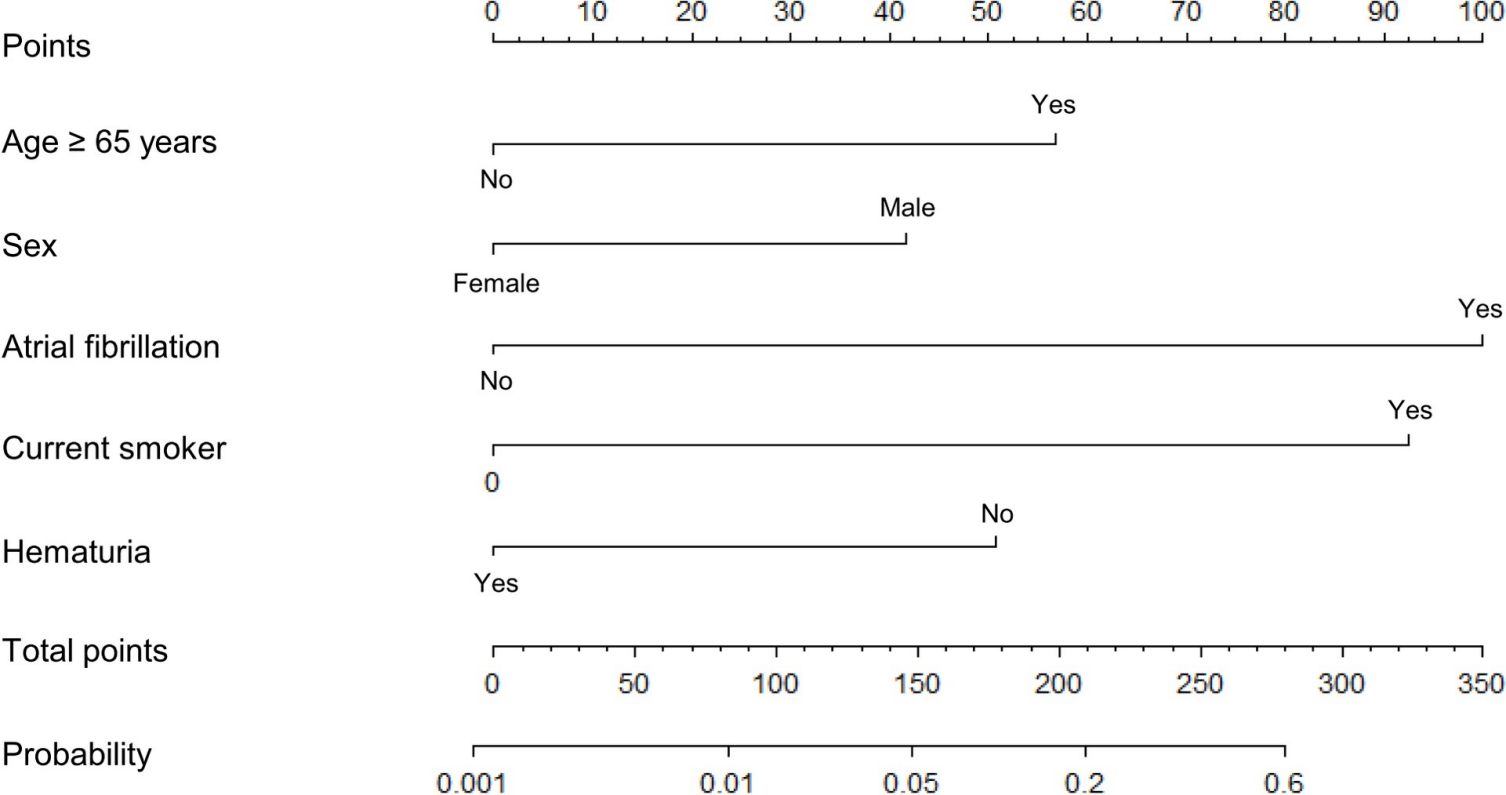
Nomogram to predict renal infarction in patients with flank pain.

The nomogram in Fig 3 was prepared to predict the probability of RI using the five significant variables (age ≥ 65, male sex, current smoker, AFib, and no hematuria). The nomogram shows that AFib was the most effective factor for predicting acute RI, followed by current smoker, age ≥ 65 years, no hematuria, and male sex. Each predictor coincided with a score on a point scale axis from 0 to 100, and RI was predicted by summing the scores of each factor.

## Discussion

We identified clinical factors that could predict acute RI in patients who visited the ED with flank pain. To the best of our knowledge, this is the first study to confirm potential predictors of RI by comparing the clinical characteristics of patients with and without RI who presented to the ED with flank pain. This study shows that acute RI should be considered in patients complaining of flank pain with the following characteristics and clinical findings: age ≥ 65 years, male sex, AFib, current smoker, and no hematuria.

Approximately 70,000 patients per year visit the hospital where this study was conducted. The incidence of RI in the ED was 0.015% during our study period, which is higher than the previously reported rate of 0.004–0.007% [2, 3]. The reason why the incidence rate of RI is higher in our study is that the rate of use of CT relative to patient number in the ED has been gradually increasing over time, so the diagnostic rate of RI might have increased [18, 19]. Also, as the need for contrast CT was determined by the clinician who treated each patient, there may be a difference in the diagnostic rate of RI due to each clinician’s experience and skill level.

Previous studies have reported that 27–32% of RI patients develop long-term sequelae, such as chronic kidney disease, and 2–5% progress to end-stage renal disease [7, 8, 20]. Therefore, it is important to identify appropriate factors for predicting RI because RI can result in long-term complications.

Huang et al. reported that the majority of 38 RI patients were male, aged > 50 years, had cardiogenic factors, such as AFib, and exhibited proteinuria and elevated LDH levels [12]. Also, according to Korzets et al., who conducted a retrospective study of 11 RI patients, hematuria, an elevated LDH level, and leukocytosis were predictors of the occurrence of RI in patients complaining of flank pain with a thromboembolism risk [10]. AFib is a risk factor for secondary thromboembolism and is a major cause of RI [7, 8, 20, 21]. Old age (≥ 65 years) has been reported as a risk factor for arterial occlusion diseases, such as stroke and pulmonary embolism [22, 23]. Similar to previous studies, our study revealed that old age (≥ 65 years) and AFib are predictors of RI. Additionally, male sex, current smoker, and no hematuria were other predictors of RI.

Whether sex differences are a risk factor for vessel diseases, such as cardiovascular disease, stroke, and pulmonary embolism, remains controversial [24–26]. Some studies have suggested that male sex is associated with an increased risk of cardiovascular disease and stroke [24, 26]. However, in a study of Americans during the 2003–2011 period, the incidence of pulmonary embolism in females was high and the prognosis was poor [25]. In previous studies of RI, the proportion of male patients was approximately two- to four-fold that of female patients [3, 8, 12, 13]. In our study, the number of male patients was approximately three-fold that of female patients, as in previous studies. Multiple factors are suggested to contribute to sex differences in vascular disease, such as genetic factor, hormonal fluctuation, lifestyle and environmental influences [24, 27]. In our study, sex differences may have occurred due to factors such as smoking habitus (more prevalent in male) [27].

Current smoking is a well-known risk factor for arterial thrombosis, because arteries can be affected by nicotine and its metabolites produced by smoking [28, 29]. Smoking causes vasoconstriction during both the acute and chronic phases [30]. Also, smoking increases platelet-dependent thrombin levels, platelet aggregation, and fibrinogen concentrations, and induces a pro-thrombotic state that inhibits fibrinolysis [30]. According to previous studies, smoking is a risk factor for arterial occlusion disease, and in our study, current smoker was a risk factor for developing RI [31].

According to studies that have discussed the characteristics of RI, hematuria is a predictor of RI [12, 21]. This is because glomerular and tubular damage resulting from tissue necrosis during RI may cause hematuria. However, in our study, “no hematuria” was significantly associated with the occurrence of RI. One reason could be that urolithiasis is a common disease in the majority of patients complaining of flank pain, and hematuria is very common in patients with urolithiasis, so hematuria may not be a reliable predictor of RI [32–34]. Also, because RI-related structural kidney damage can take several hours to occur, hematuria may not be detected in the urinalysis at the initial presentation in the ED [12, 21]. Therefore, additional research is needed to determine the relationship between hematuria and RI through serial urinalysis.

According to the nomogram created to help decision-making for RI diagnosis, AFib was the most effective factor predicting acute RI (Fig 3). Each factor was scored, and the probability is indicated, thus providing a meaningful decision-making tool for predicting RI and whether to proceed with an examination by a clinician. These clinical results can be an important measure for judging whether a test should be performed on a patient with flank pain in the ED. However, external verification and additional research will be required for generalization.

Several limitations of this study should be discussed. First, as this was a single-center study, the number of RI patients was very small (only 39 RI patients) to draw solid conclusions regarding the risk factors for RI. However, RI is a relatively rare disease with a very low incidence. Therefore, a multi-center study targeting more patients will be needed in the future. Second, this study was retrospective, and there might have been selection bias. As only patients who underwent contrast-enhanced CT according to the clinician’s judgment were included, and many patients who did not undergo CT or who underwent non-contrast enhanced CT were excluded. It may create large bias and make the conclusion unreliable. Third, we were unable to study LDH, D-dimer levels, left ventricle systolic dysfunction, prosthetic valvular disease, nonbacterial thrombotic endocarditis and hypercoagulability states, as there were many missing data, which are suspected to be predictors of RI [11, 13, 35]. Fourth, although male was a risk factor for RI in our study, sex difference in vascular disease is still controversial. Further study is required to identify the sex difference in RI and mechanisms involved. Fifth, RI patients complain of abdominal pain as well as flank pain [7]. But in this study, only patients with flank pain were included. So, a large-scale, well-designed prospective study will be needed in the future to overcome these limitations.

## Conclusions

We revealed that five clinical factors, i.e., age ≥ 65 years, male sex, AFib, current smoker, and no hematuria, were predictors of RI. Our findings will help clinicians decide whether to perform contrast-enhanced CT to diagnose RI when patients present at the ED with flank pain.

## Supporting information

S1 Table Comparison of the baseline characteristics according to sex. (DOCX)

S2 Table Sex-stratified logistic regression analysis of predictors of renal infarction. (DOCX)
