# Supplementary material for: Predictors of renal infarction in patients presenting to the emergency department with flank pain: A retrospective observational study
Source: PLoS One. 2021 Dec 7;16(12):e0261054. doi: 10.1371/journal.pone.0261054 (PMC8651137; doi:10.1371/journal.pone.0261054)
Supplement: S1 Table — (DOCX) [file pone.0261054.s001.docx]

**Supporting information: Predictors of renal infarction in patients presenting to the emergency department with flank pain: A retrospective observational study**

**Sangun Nah, Sangsoo Han, Han Bit Kim, Sohyeon Chun, Sechan Kim, Seungho Woo, Ji Eun Moon, Young Soon Cho**

**S1 Table. Comparison of the baseline characteristics according to sex.**

|  | **Male  (n = 927)** | **Female (n = 1204)** | ***p*-value** |
| --- | --- | --- | --- |
| Age, years | 49.8 ± 14.3 | 51.1 ± 16.6 | 0.06 |
| Age≥65 (%) | 227 (24.5) | 365 (30.3) | <0.01* |
| BMI, kg/m^2^ | 25.1 ± 3.7 | 23.5 ± 4.3 | <0.01 |
| Vital signs |  |  |  |
| Systolic BP, mmHg | 138.4 ± 20.1 | 130.7 ± 20.6 | <0.01 |
| Diastolic BP, mmHg | 84.7 ± 13.6 | 80.4 ± 13.3 | <0.01 |
| Heart rate, beats/min | 82.1 ± 14.7 | 86.0 ± 15.8 | <0.01 |
| Respiratory rate, /min | 19.5 ± 1.2 | 19.5 ± 1.2 | 0.42 |
| Body temperature, ℃ | 36.7 ± 0.6 | 37.1 ± 0.8 | <0.01 |
| Comorbidities, n (%) |  |  |  |
| HTN | 264 (28.5) | 318 (26.4) | 0.32* |
| DM | 131 (14.1) | 169 (14.1) | >0.99* |
| MI | 31 (3.3) | 21 (1.8) | 0.03* |
| Stroke | 27 (2.91) | 29 (2.4) | 0.56* |
| Cancer | 66 (7.1) | 106 (8.8) | 0.18* |
| AFib | 18 (1.9) | 24 (2.0) | >0.99* |
| Current smoker, n (%) | 99 (10.7) | 62 (5.2) | <0.01* |
| Symptoms, n (%) |  |  |  |
| Nausea | 169 (18.2) | 279 (23.2) | <0.01* |
| Vomiting | 58 (6.3) | 136 (11.3) | <0.01* |
| Diarrhea | 22 (2.4) | 51 (4.2) | 0.03* |
| Laboratory findings |  |  |  |
| eGFR, mL/min/1.73m^2^ | 69.3 ± 15.6 | 69.1 ± 17.4 | 0.75 |
| White blood cells, ×10^3^/mm^3^ | 9.7 ± 4.0 | 9.9 ± 5.0 | 0.36 |
| Platelet, ×10^3^/mm^3^ | 225.9 ± 65.9 | 248.4 ± 99.9 | <0.01 |
| AST, U/L | 23 [18–31] | 20 [17–26] | <0.01 |
| ALT, U/L | 25 [17–42] | 16 [12–25] | <0.01 |
| CRP, mg/dL | 0.2 [0.1–0.7] | 0.34 [0.1–2.9] | <0.01 |
| Pyuria, n (%) | 79 (8.5) | 365 (30.3) | <0.01* |
| Hematuria, n (%) | 422 (45.5) | 672 (55.8) | <0.01* |
| Renal infarction | 29 (3.1) | 10 (0.8) | <0.01* |

Note: Values are presented as the means ± standard deviations, medians [interquartile ranges], or numbers (proportions). *Pearson’s χ^2^ test. Pyuria is defined as 10 or more white blood cells per high power field (HPF). Hematuria is defined as five or more erythrocytes per HPF.

Abbreviations: BMI, body mass index; BP, blood pressure; HTN, hypertension; DM, diabetes mellitus; MI, myocardial infarction; AFib, atrial fibrillation; eGFR, estimated glomerular filtration rate; AST, aspartate transaminase; ALT, alanine transaminase; CRP, C-reactive protein; RI, renal infarction
