# Supplementary material for: Predictors of renal infarction in patients presenting to the emergency department with flank pain: A retrospective observational study
Source: PLoS One. 2021 Dec 7;16(12):e0261054. doi: 10.1371/journal.pone.0261054 (PMC8651137; doi:10.1371/journal.pone.0261054)
Supplement: S2 Table — (DOCX) [file pone.0261054.s002.docx]

**Supporting information: Predictors of renal infarction in patients presenting to the emergency department with flank pain: A retrospective observational study**

**Sangun Nah, Sangsoo Han, Han Bit Kim, Sohyeon Chun, Sechan Kim, Seungho Woo, Ji Eun Moon, Young Soon Cho**

**S2 Table. Sex-stratified logistic regression analysis of predictors of renal infarction.**

|  | **Male** | | | | **Female** | | | |
| --- | --- | --- | --- | --- | --- | --- | --- | --- |
|  | **OR** | **95% CI** | **p-value** | **VIF** | **OR** | **95% CI** | **p-value** | **VIF** |
| Age (≥ 65 years) | 1.305 | 0.524-3.252 | 0.567 | 1.288 | 1.187 | 0.177-7.962 | 0.86 | 1.356 |
| Comorbidities |  |  |  |  |  |  |  |  |
| HTN | 1.091 | 0.436-2.728 | 0.852 | 1.347 | 4.455 | 0.793-25.042 | 0.09 | 1.106 |
| MI | 1.939 | 0.505-7.453 | 0.335 | 1.309 | 2.212 | 0.173-28.26 | 0.541 | 1.229 |
| Stroke | 1.729 | 0.404-7.406 | 0.461 | 1.319 | 0.84 | 0.072-9.881 | 0.89 | 1.073 |
| Afib | 4.394 | 1.036-18.645 | 0.045 | 1.152 | 38.016 | 7.972-181.273 | <0.001 | 1.259 |
| Current smoker | 3.623 | 1.513-8.673 | 0.004 | 1.052 | 2.268 | 0.182-28.299 | 0.525 | 1.037 |
| Laboratory findings |  |  |  |  |  |  |  |  |
| eGFR (< 60 mL/min/1.73 m^2^) | 1.71 | 0.688-4.250 | 0.248 | 1.266 | 4.905 | 0.821-29.295 | 0.081 | 1.175 |
| Hematuria | 0.174 | 0.064-0.474 | <0.001 | 1.033 | 0.785 | 0.181-3.406 | 0.746 | 1.069 |

Abbreviations: OR, odds ratio; CI, confidence interval; VIF, variance inflation factor; HTN, hypertension; MI, myocardial infarction; AFib, atrial fibrillation; eGFR, estimated glomerular filtration rate
